# Structural insights into the activation of neurokinin 2 receptor by neurokinin A

**DOI:** 10.1038/s41421-022-00437-8

**Published:** 2022-07-26

**Authors:** Wenjing Sun, Qingning Yuan, Huanhuan Zhang, Fan Yang, Shenglong Ling, Yifan Luo, Pei Lv, H. Eric Xu, Changlin Tian, Wanchao Yin, Pan Shi

**Affiliations:** 1 The First Affiliated Hospital of USTC, School of Life Sciences, Division of Life Sciences and Medicine, Joint Center for Biological Analytical Chemistry, Anhui Engineering Laboratory of Peptide Drug, Anhui Laboratory of Advanced Photonic Science and Technology, University of Science and Technology of China, Anhui Hefei, China; 2 The CAS Key Laboratory of Receptor Research, Shanghai Institute of Materia Medica, Chinese Academy of Sciences, Shanghai, China; 3 High Magnetic Field Laboratory, Chinese Academy of Sciences, Anhui Hefei, China; 4 Zhongshan Institute for Drug Discovery, Shanghai Institute of Materia Medica, Chinese Academy of Sciences, Zhongshan, Guangdong China

**Keywords:** Cryoelectron microscopy, Cell signalling

Dear Editor,

Tachykinins are a family of neuropeptides distributed in the mammalian central and peripheral nervous systems^1^. The tachykinin peptides substance P (SP), neurokinin A (NKA) and neurokinin B (NKB) share a conserved C-terminal motif (-Phe-X-Gly-Leu-Met-NH_2_, X-Phe/Val), which is critical for the activation of tachykinin receptors (NK1R, NK2R and NK3R)^1^. NKA preferentially activates the neurokinin 2 receptor (NK2R) coupled to Gα_q_^2^, while SP and NKB preferentially bind the tachykinin receptors NK1R and NK3R, respectively^1^. All three tachykinins are capable of behaving as full agonists at all tachykinin receptor types^1^.

NK2R universally exists in the central and peripheral nervous systems^3^. Activation of NK2R by NKA is associated with diverse biological responses, such as intestinal motor functions, smooth muscle contraction, inflammation and asthma^3,4^. Given the important physiological functions of NK2R, it has long been considered an attractive therapeutic target in multiple diseases, ranging from asthma, depression and anxiety disorders, and irritable bowel syndrome (IBS). However, only five NK1R antagonists have been approved for use in humans, and no drugs targeting NK2R have been developed yet^5^. The inherent promiscuity of the tachykinin system and an inadequate understanding of the activation mechanism of NKRs may hamper the NKR-targeted drug design^1^.

Herein, to explore the mechanism of NK2R activation by NKA, we implemented the NanoBiT tethering strategy^6^ to assemble NKA-bound NK2R–G_q_ complex. The cryo-EM structure of the NKA-bound NK2R–G_q_ complex was determined at 2.7 Å resolution (Fig. 1a, b; Supplementary Figs. S1, S2 and Table S1). The electron density for NKA in the agonist-binding pocket of the NK2R–G_q_ complex is well defined (Fig. 1c, d).

**Fig. 1:**
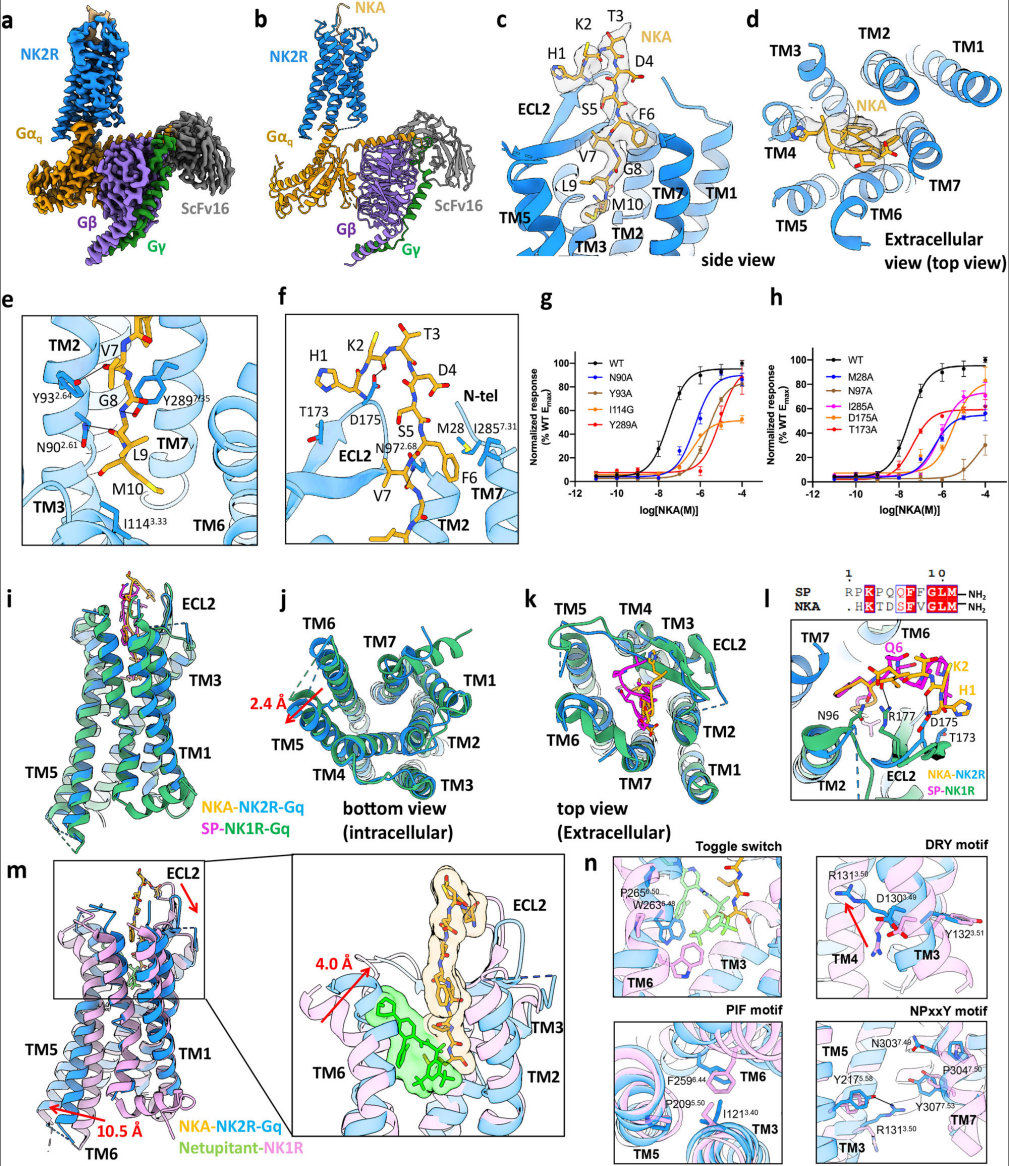
Structural insights into the activation of NK2R by NKA. **a**, **b** Cryo-EM density map (**a**) and ribbon diagram representation (**b**) of NK2R–G_q_/NKA complex. **c**, **d** Side and top views of the ligand-binding pocket of NK2R–G_q_/NKA complex. **e** The polar interactions between the C-terminus of NKA and TMDs of NK2R. **f** Interactions between the N-terminus of NKA and the extracellular surface of NK2R. **g**, **h** Ca^2+^ accumulation analysis of wild-type NK2R and mutants that interact with the C- (**g**) and N-termini (**h**) of NKA. **i**–**k** The side (**i**), bottom (**j**) and top (**k**) views of the comparison between the NKA-bound NK2R and the SP-bound NK1R structures. **l** Superimposition of the ECL2s of the NKA-bound NK2R and the SP-bound NK1R. **m** The comparison of the NKA-bound active-state NK2R (blue) and the antagonist netupitant-bound inactive-state NK1R (green) structures. **n** Conformational changes of toggle switch, PIF motif, DRY motif and NPxxY motif after NK2R activation upon binding of NKA.

NKA is a linear peptide consisting of 10 amino acids with an amidated methionine at its C-terminus. The structure shows that NKA interacts with residues in both the transmembrane region and the extracellular loops (ECLs) of NK2R (Fig. 1c, d), which is similar to the binding mode of SP-bound NK1R. The conserved C-terminus of NKA inserts into the central cavity of the transmembrane domain (TMD) of NK2R. A polar interaction network between the conserved C-terminal residues of NKA and the residues of TM2/3/7 of NK2R was observed. The side chains of Y93^2.64^ (Ballesteros-Weinstein numbering^7^) and N90^2.61^ of TM2 interact with the carbonyl oxygen of Val7 and Leu9 of NKA, respectively (Fig. 1e). N97^2.68^ at the extracellular end of TM2 can form a polar interaction with the backbone carbonyl oxygen of Val7. Additionally, Y289^7.35^ of TM7 can form a hydrogen-bond interaction with Gly8 of NKA (Fig. 1e, f). Moreover, I114^3.33^ of TM3 can form a hydrophobic interaction with Met10 to further stabilize the C-terminus of NKA. Alanine substitution markedly decreased the activation of NK2R by NKA in Ca^2+^ mobilization assays (Fig. 1g, h; Supplementary Table S2).

The N-termini of tachykinins are critical regions associated with their subtype selectivity^8^. The structure shows that the N-terminus of NKA is mainly stabilized by ECL2 of the receptor (Fig. 1f). Residue D175 of ECL2 can form a salt bridge with the main-chain carbonyl oxygen of Lys2 of NKA. The hydrophobic interactions between Phe6 in NKA and M28 at the N-terminus of NK2R and I285^7.31^ of TM7 also stabilize the ligand. Mutagenesis experiments further supported these observations, as substitution of these residues with alanine substantially attenuated the potency of NKA (Fig. 1h; Supplementary Table S2).

Superimposition of the complex structures of NK2R–G_q_/NKA and NK1R–G_q_/SP (PDB: 7RMG) revealed the high overall homology of the structures, with a root mean standard deviation (r.m.s.d.) of 0.83 Å (Fig. 1i). The conformations of the transmembrane helices of NK1R and NK2R are highly similar in the intracellular side, except that the C-terminus of TM5 of NK2R is further shifted outward by 2.4 Å compared to the position of NK1R when measured by L224^5.65^ of NK2R and L223^5.65^ of NK1R (Fig. 1j). The difference between these two structures mainly comes from the extracellular domains, especially the conformations of ECL2s (Fig. 1k). Sequence alignment of NKA and SP shows high conservation of their C-terminal sequences but deviation in their N-terminal sequences (Fig. 1l). Therefore, the N-terminal sequences may mainly account for the stronger potency and efficacy of NKA than SP to activate NK2R (Supplementary Fig. S3). In the structure of the NKA-bound NK2R–G_q_ complex, ECL2 mainly interacts with the first two N-terminal residues His1 and Lys2 of NKA, while in the recently reported SP-bound NK1R–G_q_ structure, R177 of ECL2 forms an extended hydrogen-bond interaction with the side chain of N96^2.68^ and the main-chain carbonyl oxygen of Gln6 of SP^8,9^ (Fig. 1l). The amino acid at position 1 of NKA involves the replacement of the proline in SP with histidine, allowing it to interact with T173 on ECL2, which is supported by alanine substitution and Ca^2+^ mobilization assay (Fig. 1h; Supplementary Table S2). This interaction further enables D175 to interact with the main-chain carbonyl of lysine at position 2 of NKA. The interaction between the corresponding residue Lys3 in SP and ECL2 of NK1R was not observed.^8^ Therefore, the interactions between the N-terminal residues of tachykinin play an important role in receptor subtype selectivity.

The structure of NK1R in an inactive state bound to the antagonist netupitant (PDB: 6HLP) was adopted as a reference to obtain structural features of NK2R activation (Fig. 1m; Supplementary Fig. S4). Structure comparison revealed that the agonist and antagonist have different orthosteric binding sites that overlap at the C-terminus of NKA (Fig. 1m). Because of the different occupancies of the agonist and antagonist, the extracellular end of TM6 undergoes obvious conformational changes. In the structure of NKA-bound NK2R, the extracellular end of TM6 is shifted inward by 4.0 Å compared to its position in the inactive-state NK1R (when measured at G273^6.58^ of NK2R and P271^6.58^ of NK1R) (Fig. 1m). Meanwhile, ECL2 also moves down to adapt to binding NKA. The most representative conformational change in the intracellular side is expansion of the intracellular end of TM6 by 10.5 Å relative to the inactive NK1R (when measured at N238^6.23^ of NK2R and Y236^6.23^ of NK1R) to accommodate the α5 helix of the G protein (Fig. 1m).

Moreover, a cascade of structural rearrangements of highly conserved motifs that facilitate receptor activation was observed in the NK2R receptor (Fig. 1n). Differences in the agonist- and antagonist-binding pockets further led to conformational changes in the “toggle switch”. The angular change in P265^6.50^ and the deflection of W263^6.48^ caused the intracellular end of TM6 of NKA-bound NK2R to expand outward. This conformational change further led to opening of the PIF motif and activation of the receptor for G protein binding. Another hallmark conformational change was found to occur in the DRY motif of which the side chain of R131^3.50^ swings up to form an electrostatic interaction with Y217^5.58^ of TM5. A previous study showed that after NK1R activation, E78^2.50^ moves down and interacts with N301^7.49^ and N50^1.50^ to regulate the electrostatic interaction between Y305^7.53^ of the motif NPxxY and R130^3.50^.^8^ However, the structural comparison showed that E78^2.50^ was replaced by D79^2.50^ in NK2R. In addition, N303^7.49^ moved upwards after receptor activation to reshape the electrostatic interaction with D79^2.50^, rendering Y307^7.53^ unable to interact with R131^3.50^ (Fig. 1n; Supplementary Fig. S5). Despite the different patterns of NPxxY motifs in NK1R and NK2R, neither of them undergoes an inward movement at the intracellular end of TM7, unlike other class A family GPCRs (Supplementary Fig. S6)^10,11^. This may reveal the different activation of the tachykinin receptor family.

In summary, the cryo-EM structure of the NK2R–G_q_/NKA complex was determined at a resolution of 2.7 Å. Comparing the structure of NK2R–G_q_/NKA with the previously reported structure of NK1R–G_q_/SP, shows that the residue Lys2 of NKA may play a critical role by interacting with ECL2 of NK2R, but no such interaction between corresponding residue Lys3 of SP with ECL2 of NK1R was observed. The position 1 residue of NKA is replaced from a proline in SP to a histidine in NKA, allowing it to interact with T173 on ECL2, which further stabilizes the binding of NKA. The differences mainly account for the preferential binding of NKA to NK2R compared to the binding of SP to NK1R. Furthermore, the intracellular surface of TM7 does not move inward but remains in an inactive conformation, which may represent the different activation of the tachykinin receptor family. Combined with structural and functional studies, our data reveal a framework for understanding the activation of NK2R by its preferential endogenous neuropeptide NKA, which will be helpful for the further development of more efficient therapies against diseases associated to NK2R.

## Supplementary information

Supplementary information

## Data Availability

The cryo-EM density maps and corresponding atomic coordinates of the NK2R–G_q_/NKA complexes have been deposited in the Electron Microscopy Data Bank and the Protein Data Bank under the accession codes of EMD-33497 and 7XWO, respectively. All data analyzed in this study are included in this paper and Supplementary information.
