# Supplementary material for: Structural insights into the activation of neurokinin 2 receptor by neurokinin A
Source: Cell Discov. 2022 Jul 26;8:72. doi: 10.1038/s41421-022-00437-8 (PMC9325979; doi:10.1038/s41421-022-00437-8)
Supplement: Supplementary file 1 — Supplementary information [file 41421_2022_437_MOESM1_ESM.pdf]

Supplementary Materials for

Structural insights into the activation of neurokinin 2 receptor by neurokinin A

Materials and Methods

Construct cloning

The optimized coding DNA for wild type *Homo sapiens* NK2R (UniProt accession: P21452) was synthesized by Genscript. And residues 1–345 of NK2R with an N-terminal thermostabilized apocytochrome b_{562}RIL (BRIL)[1] and a C-terminal LgBiT were cloned into pFastBac vector using homologous recombination (CloneExpress One Step Cloning Kit, Vazyme). Before BRIL fragment, there are HA and FLAG tags followed by a His10 (H10) tag, as well as a TEV cleavage site. The engineered $G\alpha_q$ construct was generated on the basis of mini-$G_{s/q}$71[2] with two dominant-negative mutations (corresponding to G203A and A326S) [3] to decrease the affinity of nucleotide binding. The N-terminal 1-18 amino acids and the α-helical domain of the mini-$G_{s/q}$71 were replaced by the corresponding sequences of the human $G\alpha_{i1}$, providing possible binding sites for three antibody fragments Nb35, scFv16 and Fab-G50[4,5]. The Nb35 and scFv16 were used to stabling the NK2R-G_q complex and Fab50 did not be added in this work. Rat Gβ1 with an N-terminal His6 tag was followed by HiBiT at its C terminus. The engineered $G\alpha_q$, Gβ1 and bovine Gγ2 were cloned into the pFastBac vector (Invitrogen).

Expression and purification of Nb35

Nb35[6] with a C-terminal His6 tag was expressed in *Escherichia coli* BL21 (DE3) bacteria, and cultured in LB medium with 50 $\mu g ml^{-1}$ ampicillin to an optical density (OD_{600}) value of 0.6 -1.0 at 37 °C, 180 r.p.m. The IPTG (1mM) was added to induce Nb35 expression at 27 °C, 180 r.p.m for 8 h. The *E. coli* bacteria were then collected by centrifugation (4,000 r.p.m., 20 min) and disrupted in 20 mM HEPES, pH7.4, 100 mM NaCl, 10% glycerol and 1mM PMSF. Cell pellets were removed by centrifugation (8,000 r.p.m., 30 min) and the supernatant was purified by nickel affinity chromatography (Ni Smart Beads 6FF, Smart Life Sciences). The resin was washed with 30 column

volumes of buffer containing 20 mM HEPES pH7.4, 100 mM NaCl, 25 mM imidazole, 10% glycerol, and the eluted protein was collected with buffer containing 20 mM HEPES pH 7.4, 100 mM NaCl, 200 mM imidazole and 10% glycerol. The eluted Nb35 was concentrated and subjected to a HiLoad 16/600 Superdex 75 column (GE Healthcare) pre-equilibrated with buffer containing 20 mM HEPES pH 7.4 and 100 mM NaCl. The monomeric fractions were collected and stored with 30% (v/v) glycerol at −80˚C for future use.

Expression and purification of NK2R–G_q complex

High 5 cells were infected with viruses of the receptor (NK2R), $G\alpha_q$, Gβ1, Gγ2 and scFv16 in the ratio of 1:1:1:1:1 for 48 h at 27 ˚C. The cell pellets were lysed by dounce homogenization in 20 mM HEPES pH7.4, 100 mM NaCl, 10 mM $MgCl_2$, 5 mM $CaCl_2$, 10% glycerol, EDTA-free protease inhibitor cocktail (TargetMol) and 10 μM peptide (NKA, MCE). The supernatant was then centrifuged at 30,000 r.p.m. for 30 min to collect the membrane. The washed membrane was re-suspended in 20 mM HEPES pH7.4, 100 mM NaCl, 10 mM $MgCl_2$, 5 mM $CaCl_2$, 10% glycerol, 10 μM peptide (NKA, MedChemExpress), 25 $mUml^{-1}$ apyrase (Sigma-Aldrich), 100 μM TCEP (Sigma-Aldrich), EDTA-free protease inhibitor cocktail and 20 μgml^{-1} Nb35, and incubated at room temperature for 1.5 h. After incubation, 0.5% (w/v) lauryl maltose neopentylglycol (LMNG, Anatrace) and 0.1% (w/v) cholesteryl hemisuccinate (CHS, Anatrace) were used for solubilization at 4˚C for 3 h. The supernatant was collected by centrifugation at 30,000 r.p.m. for 30 min and then incubated with FLAG resin (Smart-Lifesciences) at 4˚C overnight. The resin was collected by centrifugation at 500 g for 10 min, loaded onto a gravity flow column and washed with 20 column volumes of buffer containing 20 mM HEPES pH7.4, 100 mM NaCl, 10 mM $MgCl_2$, 5 mM $CaCl_2$, 10% glycerol, 100 μM TCEP, 10 μM peptide NKA, 0.1% (w/v) lauryl maltose neopentylglycol (LMNG, Anatrace) and 0.02% (w/v) CHS. The detergent of washing buffer was then displaced by 0.03% (w/v) LMNG, 0.01% (w/v) glyco-diosgenin (GDN, Anatrace) and 0.008% (w/v) CHS for 20 column volumes washing. The attached protein complex was then eluted in buffer containing 20 mM HEPES pH7.4, 100 mM NaCl, 10 mM $MgCl_2$, 5 mM $CaCl_2$, 10% glycerol, 100 μM TCEP, 10 μM peptide NKA, 200 μg/mL FLAG peptide (Bankpeptide Biological Technology) and concentrated with an Amicon Ultra Centrifugal Filter (MWCO 100 kDa) and loaded onto a Superdex 200 10/300 GL column (GE Healthcare) with running buffer containing 20 mM HEPES

pH7.4, 100 mM NaCl, 2 mM $MgCl_2$, 100 μM TCEP, 25 μM peptide, 0.00075% (w/v) LMNG, 0.00025% (w/v) GDN and 0.0002% (w/v) CHS. The fractions of monomeric protein complex were collected and concentrated with an Amicon Ultra Centrifugal Filter (MWCO 100 kDa) by 30-50 fold for sample preparation and detection by cryo-EM.

Cryo-EM grid preparation and data collection

For cryo-EM grid preparation of the NKA-NK2R-G_q complex, 3 μL of purified protein (13 mg/ml) was loaded onto a glow-discharged holey carbon grid (Quantifoil, Au300 R1.2/1.3) using a Vitrobot chamber (FEI Vitrobot Mark IV). Cryo-EM images were collected by a Titan Krios G4 at 300 KV accelerating voltage equipped with a Gatan K3 direct electron detector at Advanced Center for Electron Microscopy at Shanghai Institute of Materia Medica, Chinese Academy of Sciences. Micrographs were recorded with a pixel size of 0.824 Å. In total 5,329 movies were obtained at a dose of 50 electron per $Å^2$ for 36 frames. The defocus range of this dataset was -0.8 μm to -2 μm.

Cryo-EM data processing three-dimensional reconstruction

Cryo-EM data of the NKA-NK2R-G_q complexes were processed using RELION v.3.1.0[7]. Initial contrast transfer function (CTF) fitting was performed with CTFFIND -4.1[8] from cryoSPARC [9]. The auto-picking and two-dimensional (2D) classification were processed using cryoSPARC, producing 975,379 particles for further processing. With initial model, two rounds of 3D classifications were carried out. A further one round of 3D classification was conducted with a mask on the receptor, in which 246,415 particles were subjected to 3D auto-refinement and polishing. A map with an indicated global resolution of 2.74 Å at a Fourier shell correlation (FSC) of 0.143 was generated from the final 3D refinement, and subsequently post-processed by DeepEMhancer[10].

Model building and refinement

All PDB coordinates using AlphaFold2[9] were served as a starting model for building the atomic model. The G_q heterotrimer was built on the basis of the corresponding G protein of the des-Arg^{10}-kallidin-B1R-G_q (PDB 7EIB)[11] complex as a template. All model were fitted into the cryo-EM density map using Chimera[12] followed by a manual adjustment in Coot[13]. The model was refined

by Phenix[14].

Calcium mobilization assay

For calcium mobilization assay, HEK293T cells were transiently transfected with wide-type or mutant NK2R (5 μg - 8 μg optimized to make the same cell surface expression). 24 h after transfection, cells were harvested and plated into poly-D-lysine coated 96-well black wall/clear bottom plates (60,000 cells per well) and incubated overnight at 37°C and 5% CO_2. On the day of assay, cells were washed with fresh calcium buffer (HBSS Gibco 14025 supplemented with 20 mM HEPES, 0.1% BSA and 2.5 mM probenecid; pH 7.4) and then loaded with 2 μM Fluro-4 AM (Thermo Fisher Scientific, F14201) in calcium buffer for 45 min at 37 °C. The plate was washed once and added with 50 μL calcium buffer per well before measuring. Calcium mobilization was measured using FLIPR (Fluorescent Image Plate Reader, Molecular Devices), fluorescence was measured at 1-s intervals for 310 s. After establishing baseline fluorescence in the first ten seconds, cells were stimulated with increasing concentrations of the NKA. The maximal change in fluorescence after agonist addition was quantitated.

Cell surface expression level

Cell surface expression level was determined by flow cytometry. HEK293T cells were seeded in six-well plates in DMEM (Gibco, Thermo Fisher Scientific) medium supplemented with 10% v/v FBS at 37°C and 5% CO2. After 24 h, the cells were transiently transfected with wild-type NK2R or mutants using Lipofectamine 3000 (Invitrogen). After 24 h transfection, cells were washed twice with 1mL PBS (Gibco, 10010023) containing 3% BSA. The cells were then pelleted and resuspended in 200 μL PBS containing 3% BSA and 1μL PE-anti-DYKDDDDK tag (BioLegend, Cat: 637310). After incubation in a 4°C and dark environment for 30 minutes, the cells were washed twice with PBS and the expression level were detected by flow cytometry (BD Biosciences). The expression level of All mutants was normalized to the expression of wild-type NK2R. Each mutant was performed in three independent experiments.

Statistics

Statistical analyses were performed on at least three individual data sets analyzed by GraphPad prism. Data are means ± SEM from at least three independent experiments performed in technical triplicate. For calcium mobilization assay, data were normalized and analyzed using nonlinear curve fitting for the log (agonist) versus response (three parameters) curves.

Supplementary Reference：

1. Chun, E. et al. Fusion Partner Toolchest for the Stabilization and Crystallization of G Protein-Coupled Receptors. *Structure* **20**, 967–976 (2012).
2. Nehmé, R. et al. Mini-G proteins: Novel tools for studying GPCRs in their active conformation. *PLoS ONE* **12**, e0175642 (2017).
3. Liu, P. et al. The structural basis of the dominant negative phenotype of the Gαi1β1γ2 G203A/A326S heterotrimer. *Acta Pharmacol Sin* **37**, 1259–1272 (2016).
4. Maeda, S., Qu, Q., Robertson, M. J., Skiniotis, G. & Kobilka, B. K. Structures of the M1 and M2 muscarinic acetylcholine receptor/G-protein complexes. *Science* **364**, 552–557 (2019).
5. Kang, Y. et al. Cryo-EM structure of human rhodopsin bound to an inhibitory G protein. *Nature* **558**, 553–558 (2018).
6. Rasmussen, S. G. F. et al. Crystal structure of the β2 adrenergic receptor–Gs protein complex. *Nature* **477**, 549–555 (2011).
7. Zivanov, J., Nakane, T. & Scheres, S. H. W. Estimation of high-order aberrations and anisotropic magnification from cryo-EM data sets in *RELION*-3.1. *IUCrJ* **7**, 253–267 (2020).
8. Rohou, A. & Grigorieff, N. CTFFIND4: Fast and accurate defocus estimation from electron micrographs. *Journal of Structural Biology* **192**, 216–221 (2015).
9. Senior, A. W. et al. Improved protein structure prediction using potentials from deep learning. *Nature* **577**, 706–710 (2020).
10. Sanchez-Garcia, R. et al. DeepEMhancer: a deep learning solution for cryo-EM volume post-processing. *Commun. Biol.* **4**, 874 (2021).
11. Yin, Y.-L. et al. Molecular basis for kinin selectivity and activation of the human bradykinin receptors. *Nat. Struct. Mol. Biol.* **28**, 755–761 (2021).
12. Goddard, T. D., Huang, C. C. & Ferrin, T. E. Visualizing density maps with UCSF Chimera. *Journal of Structural Biology* **157**, 281–287 (2007).
13. Emsley, P. & Cowtan, K. *Coot*: model-building tools for molecular graphics. *Acta Crystallogr D Biol Crystallogr* **60**, 2126–2132 (2004).
14. Adams, P. D. et al. *PHENIX*: a comprehensive Python-based system for macromolecular structure solution. *Acta Crystallogr D Biol Crystallogr* **66**, 213–221 (2010).

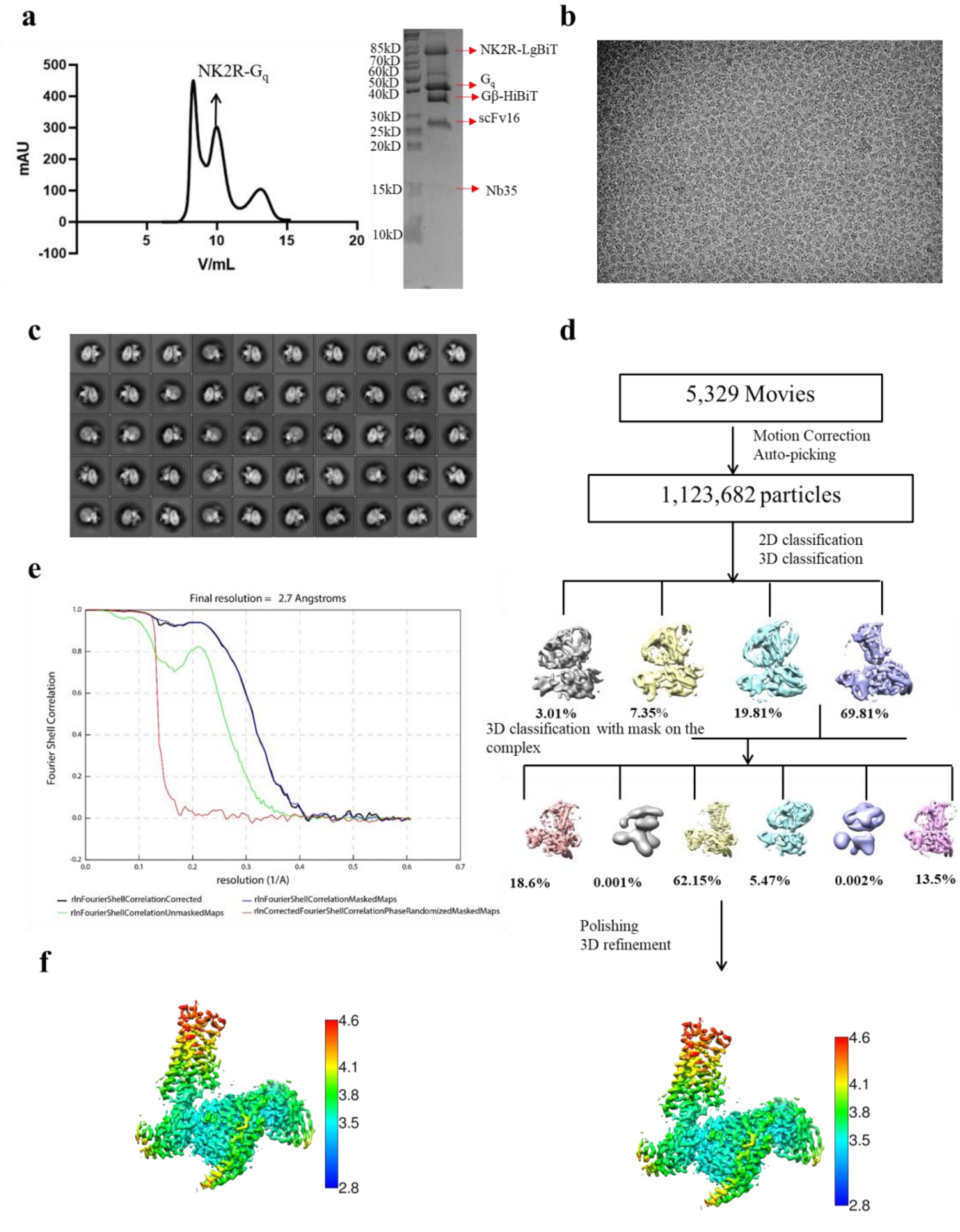

Fig. S1 Purification and cryo-EM data processing of NKA bound NK2R-G_q complex.

a Size-exclusion chromatography profile and SDS-PAGE analysis of NK2R-G_q complex bound with NKA. **b** Representative micrograph after motion correction and dose weighting. **c** 2D class averages of NK2R-G_q complex bound with NKA. **d** Flow chart of cryo-EM data processing using cryoSPARC. **e** Gold-standard FSC validation curves from cryoSPARC. **f** Density map of NKA-NK2R-G_q complex colored by local resolution estimation.

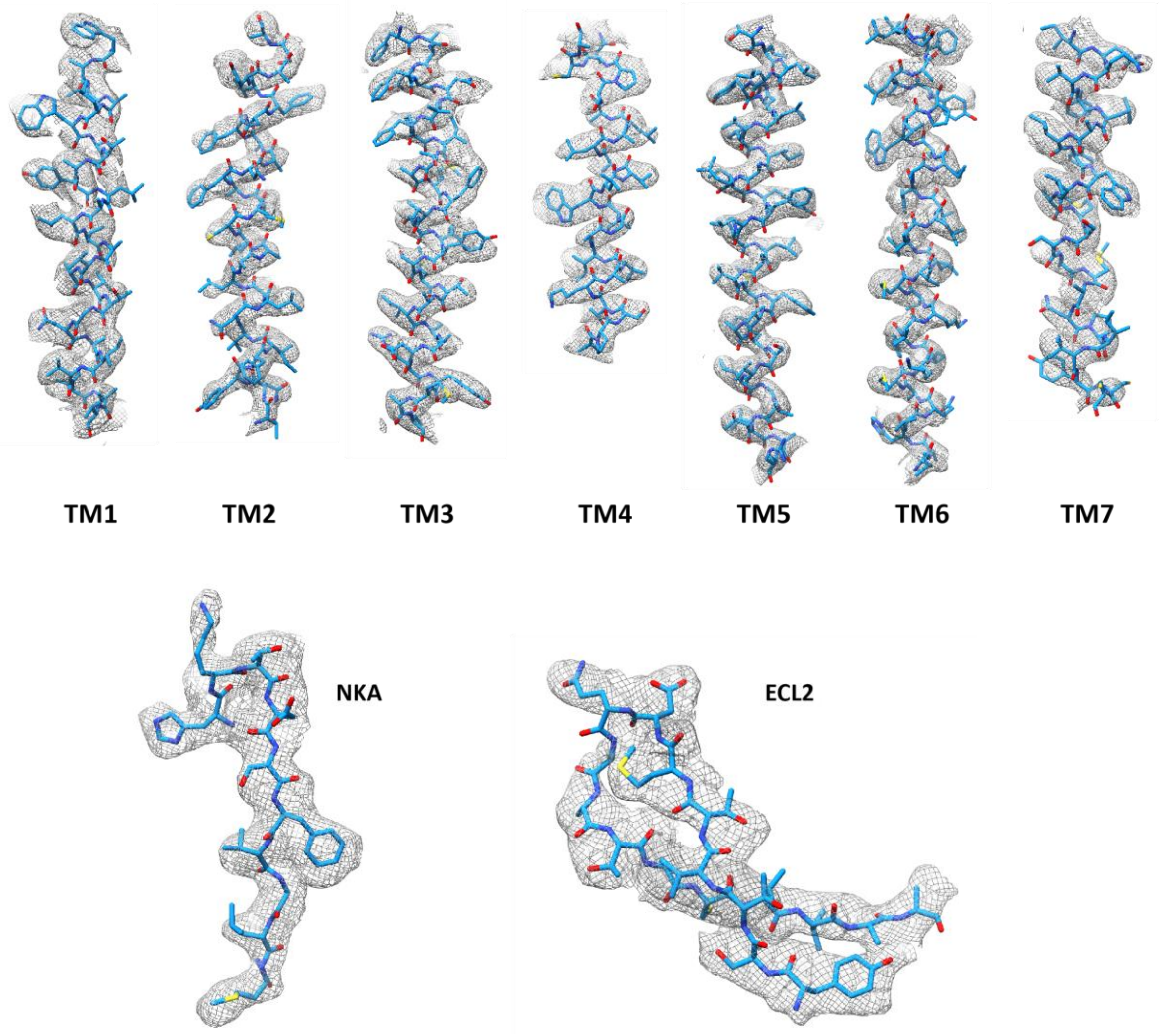

Fig. S2 Cryo-EM densities of representative segments of NKA bound NK2R-G_q complex.

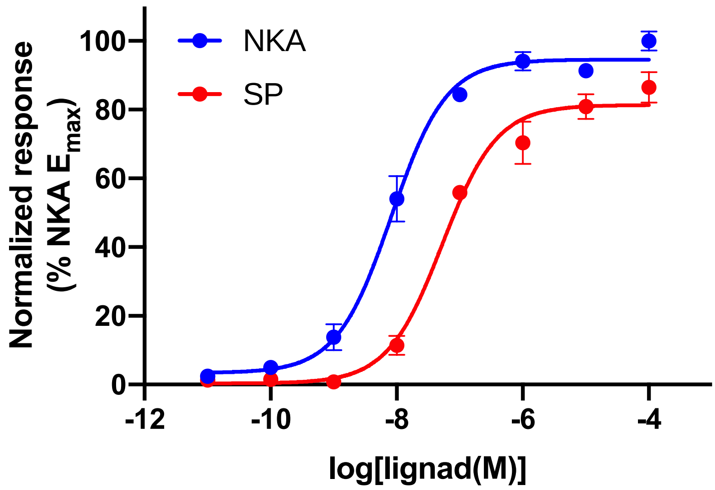

Fig. S3 Ca^{2+} mobilization assay of NK2R activation by SP or NAK. Data are expressed as means ± SEM of three independent experiments conducted in triplicate.

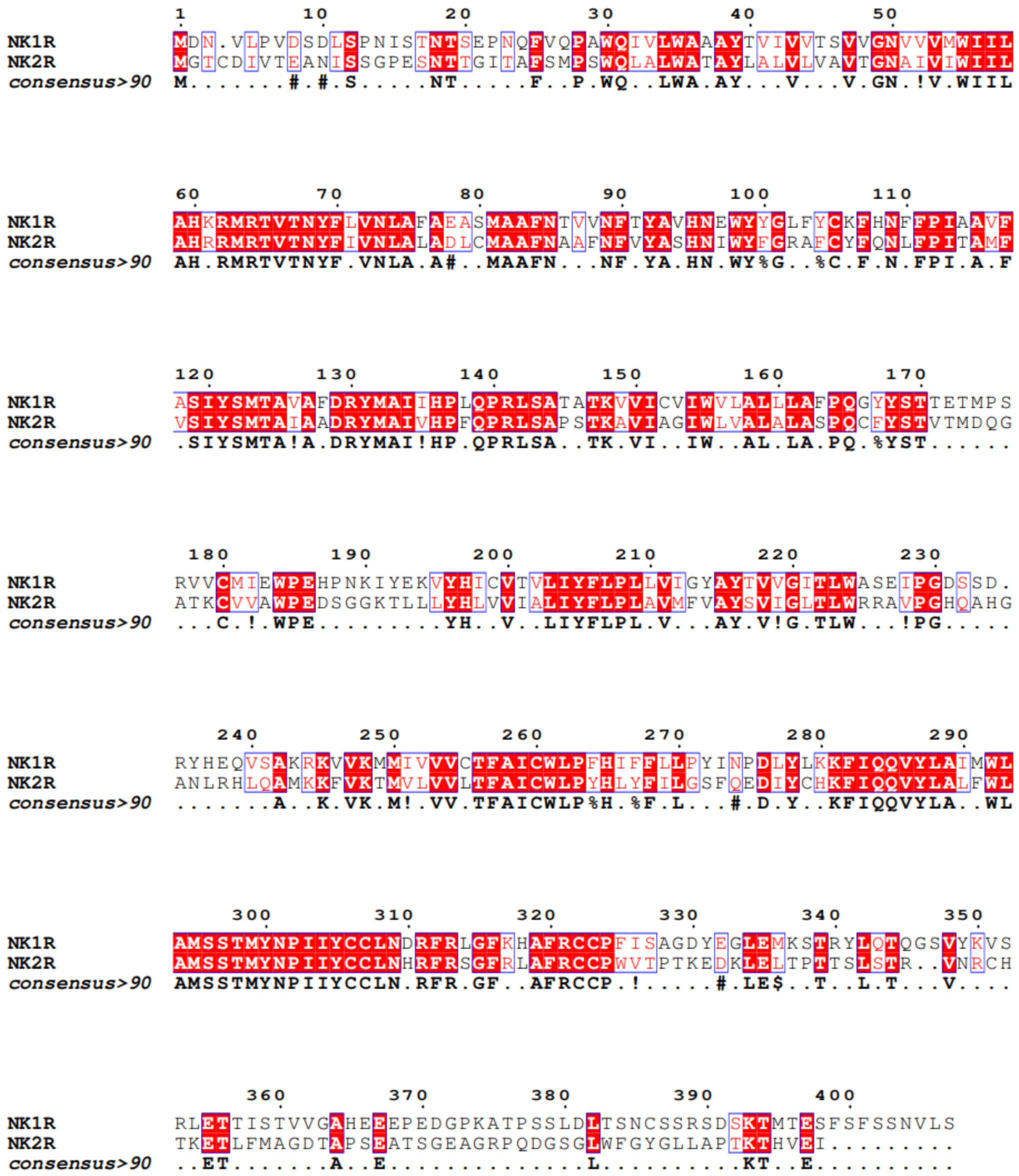

Fig. S4 Sequence alignment of NK1R and NK2R.

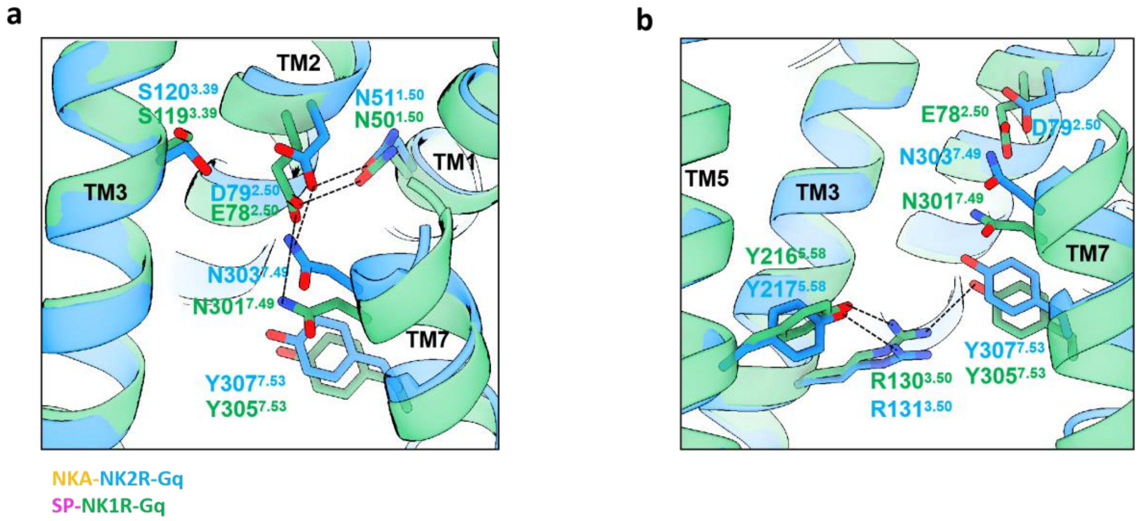

Fig. S5 Structure comparison of the NPxxY motif of NKA-bound NK2R-G_q and SP-bound NK1R-G_q complexes (PDB:7RMG).

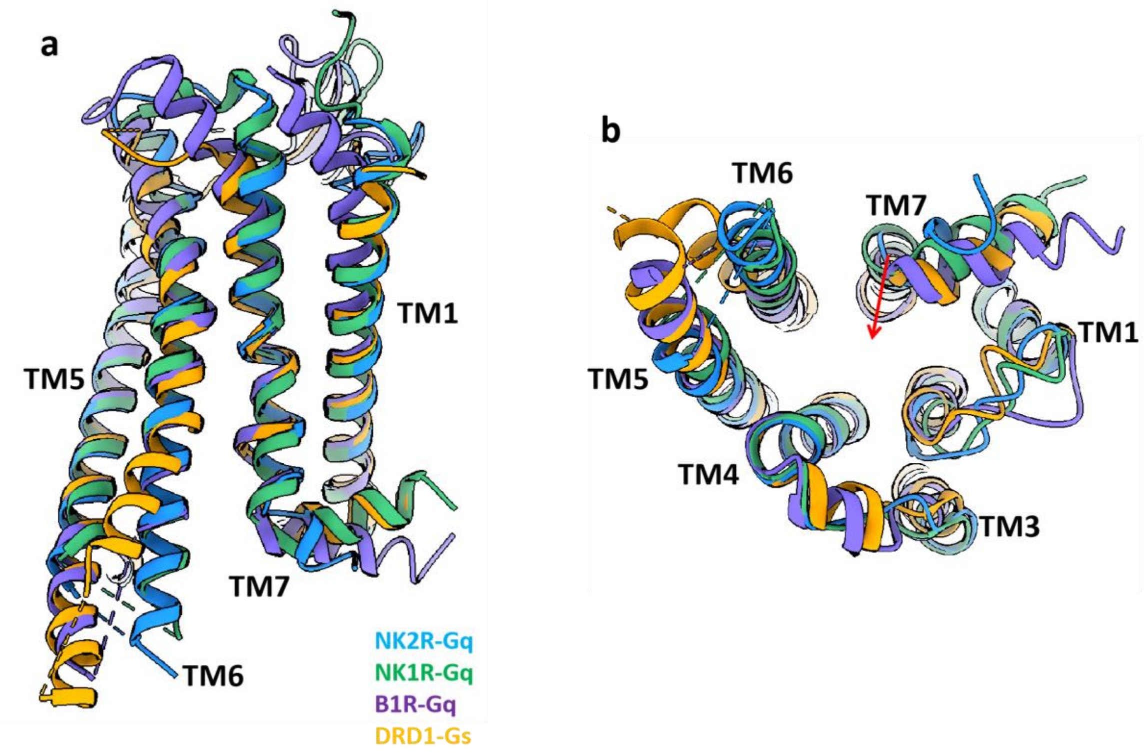

Fig. S6 The comparison of TM7 conformation between NK1R-Gq (PDB:7RMG), NK2R-G_q with B1R-G_q (PDB:7EIB) and DRD1-G_s (PDB:7CKZ) structures.

Table S1 | Statistics of cryo-EM data collection, 3D reconstruction and model refinement.

**Data Collection**	
**Protein**	NKA-NK2R
**Microscope**	FEI Titan Krios
**Voltage (kV)**	300
**Detector**	Gatan K3 Summit
**Detector mode**	Super
**Pixel size (Å)**	0.824
**Defocus range (μm)**	-0.8 ~ -2.0
**Electron dose (e^-/Å^2)**	50
**Frames per image**	36
**Exposure time (s)**	2.35
**Number of images**	5,329
**3D reconstruction**	
**Final Particle number**	610,257
**Symmetry**	C1
**Overall resolution (Å)**	2.7
**Model refinement**	
**Model composition**	
**Chains**	6
**Ligands**	LIG:1
**Non-hydrogen atoms**	8,933
**Protein residues**	1,139
**Bonds (RMSD)**	
**Length (Å)**	0.002
**Angles (°)**	0.535

**Ramachandran plot (%)**	
**Outliers**	0.10
**Allowed**	3.22
**Favored**	96.69
**Rotamer outliers (%)**	0.76
**MolProbity score**	1.55
**Clash score**	6.21

Table S2 | Ca^{2+} mobilization assay of wild-type NK2R and mutants activation by NKA. pEC_{50} values represent the negative logarithm of agonist concentration that produces half maximal response. Emax values are maximal response as percentage of wild-type NK2R response. All values are expressed as mean ± S.E.M. of three independent experiments conducted in triplicate.

Mutant	EC50(nM)	Fold shift	pEC50±S. E. M (n)	Emax±S. E. M (n) (% of WT)	Expression(% of WT)
WT	22.66	1	7.66±0.07(3)	100(3)	100
M28A	434.77	19.19	6.36±0.01(3)	58.84±2.72 (3)	104.67±15.73 (3)
N90A	374.90	16.54	6.48±0.15(3)	93.00±9.22 (3)	116.30±4.27 (3)
Y93A	3205.33	141.45	5.53±0.12(3)	84.68±6.23 (3)	88.77±8.89 (3)
N97A	n.d.	n.d.	n.d.	n.d.	81.95±22.68 (3)
I114G	606.00	26.74	6.25±0.11(3)	65.64±7.32 (3)	102.58±5.46 (3)
I285A	1149.27	50.72	5.97±0.09(3)	93.61±11.02 (3)	114.31±18.15 (3)
Y289A	9194.00	405.74	5.03±0.01(3)	87.08±4.80 (3)	93.51±2.78 (3)
D175A	6893.67	304.2	5.25±0.19(3)	82.85±11.55 (3)	107.67±11.39 (3)
T173A	134.37	5.93	6.85±0.10(3)	61.83±0.92(3)	91.27±8.95(3)
